# Selenium Compounds and Their Bioactivities: Molecular Mechanisms and Prospects for Functional Food and Therapeutic Applications

**DOI:** 10.3390/plants14172622

**Published:** 2025-08-23

**Authors:** Xue Hou, Zhiyong Wang, Mu Peng

**Affiliations:** 1 Hubei Key Laboratory of Biological Resources Protection and Utilization, Hubei Minzu University, Enshi 445000, China; hx17320056301@163.com; 2 College of Biological and Food Engineering, Hubei Minzu University, Enshi 445000, China

**Keywords:** selenium, bioactivities, antioxidant defense, functional food

## Abstract

Selenium (Se) is an essential trace element for the human body and plays a vital role in various physiological processes. Plants serve not only as a major dietary source of selenium but also as natural biofactories capable of synthesizing a wide range of organic selenium compounds. The bioavailability and toxicity of selenium are highly dependent on its chemical form, which can exert varying effects on human physiology. Among these, organic selenium species exhibit higher bioavailability, lower toxicity, and greater structural diversity. In recent years, plant-derived selenium-containing compounds—selenium-enriched proteins, peptides, polysaccharides, polyphenols, and nanoselenium—have garnered increasing scientific attention. Through a systematic search of databases including PubMed, Web of Science, and Scopus, this review provides a comprehensive overview of selenium uptake and transformation in plants, selenium metabolism in humans, and the classification, composition, structural features, and biological activities of plant-derived selenium compounds, thereby providing a theoretical basis for future research on functional foods and nutritional interventions.

## 1. Introduction

Selenium (Se) is an essential trace element for living organisms and plays a vital role in human physiological processes. It exerts a wide range of biological functions, mainly in immune and antioxidant defense, tumor and organ protection, cardiovascular regulation, and several emerging roles such as anti-ferroptosis and anti-fatigue [1,2,3]. Numerous studies have demonstrated that selenium deficiency is closely associated with various diseases, such as liver disorders, Keshan disease, osteoarthritis, kidney disease, cancer, cardiovascular diseases, acquired immunodeficiency syndrome (AIDS), and severe malnutrition [4,5,6]. Moreover, dysregulation of selenium status and selenoprotein expression has been implicated in neurological disorders, including Alzheimer’s disease, Parkinson’s disease, depression, amyotrophic lateral sclerosis (ALS), and multiple sclerosis (MS) [7]. However, the effective supplementation dose of inorganic selenium is very close to its toxic threshold, and even slight imbalance can lead to toxicity, highlighting the need for careful dosage control [8].

Among various sources, plant-derived organic selenium compounds—such as seleno-methionine (SeMet)—exhibit significantly lower toxicity and higher bioavailability than inorganic forms [9]. Because selenium cannot be synthesized endogenously by the human body nor stored for long periods, continuous dietary intake is required to maintain selenium homeostasis [10]. However, the human body’s absorption and utilization efficiency of inorganic selenium is relatively limited. Once absorbed, both organic and inorganic selenium are ultimately converted into low-molecular-weight selenium species that serve as precursors for selenoprotein synthesis [11]. These characteristics make plant-derived selenium compounds ideal dietary sources with strong potential for application in functional foods targeting anti-aging, anticancer, and other health-promoting purposes. Accordingly, this review aims to explore the critical scientific question of how plant-derived selenium compounds can serve as safe, highly bioavailable, and multifunctional dietary sources for the prevention and management of diseases associated with oxidative stress and selenium deficiency. To this end, we performed a systematic literature search using PubMed, Web of Science, and Scopus, summarizing current evidence on their biological activities, mechanisms of action, and application prospects in functional foods and nutritional interventions.

## 2. Properties of Selenium and Functions

Selenium exists in various forms in the environment, with the most abundant being selenite (SeO_3_^2−^, Se(IV)), selenate (SeO_4_^2−^, Se(VI)), and selenides (Se^2−^) and elemental selenium (Se^0^) [12]. It is primarily distributed in rocks, soils, lakes, oceans, the atmosphere, water bodies, and the biosphere [13]. However, its distribution is highly heterogeneous across different regions, and selenium deficiency is prevalent in many parts of the world, for example, in certain areas of Greece and India [10]. Selenium deficiency can lead to various systemic disorders; for example, Keshan disease—first identified in the 1930s in selenium-deficient regions of China—had incidence rates as high as 50%, and its decline has been associated with the use of selenium-fortified salt, underscoring selenium’s therapeutic potential [14]. In living organisms, selenium predominantly occurs in the form of SeMet and selenocysteine (SeCys), both of which are incorporated into selenoproteins [15]. SeMet serves as a non-specific selenium reservoir, substituting for methionine in protein synthesis when selenium intake is low [16,17]. In contrast, SeCys is a biologically active and forms an essential part of selenoproteins, which are crucial for various physiological functions [18]. To date, over 30 selenoproteins have been identified, with nearly 15 successfully purified in vitro, providing critical insights into their biological functions and associations with various diseases [10,19,20]. Selenium’s antioxidant properties protect cells from oxidative stress induced dam-age—such as lipid peroxidation, protein carbonylation, and DNA lesions—thereby lowering the risk of chronic diseases including cardiovascular disorders and certain cancers; its immunomodulatory and anti-inflammatory activities support thymic integrity, maintain lymphocyte viability, and enhance antibody production, contributing to tumor prevention; selenium and its compounds also modulate the nervous system, effectively preventing neurodegenerative diseases such as Alzheimer’s disease, Parkinson’s disease, and ALS; moreover, selenium plays preventive and therapeutic roles in thyroid function, diabetes management, male in-fertility, Keshan disease, Kashin–Beck disease, and arthritis [10].

Selenium compounds are broadly categorized into inorganic forms (e.g., selenates and selenites) and organic forms (e.g., selenopolysaccharides, selenoproteins, selenium-containing nucleic acids, alkylated selenium compounds, selenoamino acids, and their derivatives) [21]. Inorganic selenium compounds, due to their high toxicity and low bioavailability, are not used in food applications, with countries like the United States and Japan banning them from food products [22]. Acute selenium toxicity, resulting from high-dose ingestion over a short period, primarily manifests as respiratory distress, ataxia, diarrhea, vomiting, abdominal pain, and, in severe cases, death. In contrast, chronic selenium toxicity, caused by prolonged low-dose exposure, is characterized by symptoms such as fatigue, depression, garlic-like breath odor, anemia, reduced appetite, alopecia, nail shedding, hoof lesions, growth retardation, and hepatic cirrhosis [23].

Furthermore, excessive selenium intake can lead to specific toxicities, including genotoxicity, embryotoxicity, reproductive toxicity, immunotoxicity, and cytotoxicity [10,24]. Recent studies have demonstrated that the toxicity of selenium is largely dependent on its chemical form, and its toxic effects may vary according to animal species, nutritional status, and route of administration. Ruminants such as horses, cattle, and sheep, as well as livestock like pigs, are relatively more susceptible to both acute and chronic selenium intoxication, while rodent models also exhibit high sensitivity under excessive exposure [25]. Nutritional status markedly modulates selenium toxicity [26]; for instance, low-protein diets, high-sulfur feed, and compromised antioxidant defenses can exacerbate toxic effects, whereas adequate protein intake or supplementation with sulfur-containing amino acids may partially mitigate these adverse outcomes. Among administration routes, oral delivery is generally safer than parenteral injections (subcutaneous or intravenous), owing to its slower absorption rate and the facilitation of fecal excretion, which together help reduce peak blood selenium concentrations and tissue accumulation, thereby lowering the risk of acute toxicity [27]. By contrast, most organic selenium compounds are considered superior selenium sources and are often present in bioactive forms. These compounds exhibit low toxicity, minimal side effects, high biocompatibility, environmental safety, and efficient reutilization in the human body [28]. For example, selenopolysaccharides possess dual physiological functions: they not only serve as a source of metabolic energy but also effectively replenish selenium levels, making them one of the most promising selenium supplements [29].

## 3. Global Research Progress on Selenium in Plants (2000–2025)

**Figure 1:**
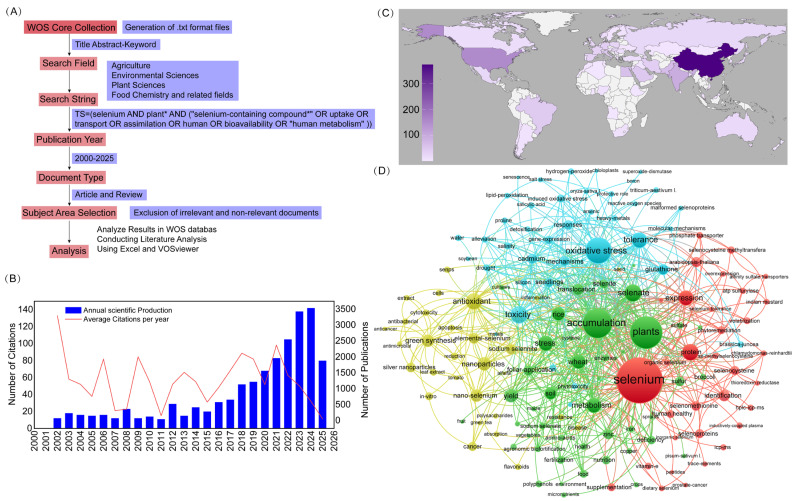
A bibliometric analysis of global research trends on selenium and selenium-containing compounds in plants (2000–2025) based on data from the Web of Science Core Collection. (**A**) Search strategy and analysis workflow, (**B**) Trends in annual scientific production and citations, (**C**) Geographic distribution of research output, and (**D**) Keyword co-occurrence network highlighting major research themes.

A comprehensive bibliometric analysis was performed to investigate global research trends related to selenium and selenium-containing compounds in plants from 2000 to 2025, based on data retrieved from the Web of Science (WOS) Core Collection (Figure 1). The literature search strategy encompassed multiple disciplines, including agronomy, plant science, environmental science, and food chemistry. A structured search query was constructed using the terms “selenium AND plant”, combined with keywords related to selenium compounds, their absorption, transport, bioavailability, and metabolic pathways in humans (Figure 1A). Only original research articles and review papers were included, while irrelevant records were excluded through manual screening. The final dataset was analyzed and visualized using Microsoft Excel and VOSviewer software (v 1.6.20).

The analysis revealed a steady increase in research output over the past two decades, with a marked acceleration observed after 2018 (Figure 1B). This trend reflects growing scientific interest in the physiological roles of selenium in plants and its nutritional significance for human health. In particular, selenium’s role in plant growth, stress regulation, and the enrichment of food crops has become a focal point of current research. From a geographical perspective, China, the United States, India, Brazil, and Germany emerged as the leading contributors to this field (Figure 1C). Keyword co-occurrence network analysis provided further insight into the research structure and thematic evolution within the field (Figure 1D). The terms “selenium” and “plants” served as central nodes, forming several tightly connected thematic clusters. These clusters represent four major research directions: (1) the mechanisms of selenium uptake, accumulation, and metabolic transformation in plants; (2) the regulatory role of selenium in plant responses to various environmental stresses; (3) the biological activity and functional characterization of selenium-enriched compounds such as selenoproteins, selenopolysaccharides, and selenopeptides; and (4) the green synthesis, application, and utilization of selenium-based materials in agriculture and food systems.

## 4. Plant Absorption, Conversion, and Metabolism of Selenium

Selenium plays a crucial role in various physiological processes, including antioxidant defense, thyroid hormone metabolism, and immune regulation through its incorporation into enzymes like glutathione peroxidase, thioredoxin reductase, and iodothyronine deiodinase [30]. Plants represent one of the primary dietary sources of selenium, making it important to understand the mechanisms of selenium uptake, transformation, and accumulation for biofortification strategies in selenium-deficient regions.

### 4.1. Mechanisms of Selenium Uptake, Transformation, and Accumulation in Plants

Selenium predominantly exists in inorganic forms in soil, with its concentration influencing the potential for plant uptake and accumulation. Plant roots primarily absorb selenium in the forms of selenate and selenite, with a generally higher affinity observed for selenate compared to selenite [31]. Soil pH also significantly affects selenium speciation and uptake [32]. In alkaline soils, selenate is the predominant form due to its high solubility and mobility, while in acidic soils, selenite dominates, leading to distinct differences in root absorption efficiency [33]. These two forms exhibit different behaviors in plant systems: selenate is more water-soluble and more readily translocated within the plant, whereas selenite is less mobile and more likely to be retained or metabolized in root tissues [34].

Selenium uptake occurs via transport proteins shared with sulfur and phosphorus, reflecting commonalities in their metabolic pathways [35]. Consequently, selenium competes with sulfur-containing compounds such as sulfide, thiosulfate, sulfite, and sulfate. Selenate, being a structural analogue of sulfate, is primarily transported through sulfate transporters, whereas selenite can be absorbed via phosphate transport channels [36]. Elevated external sulfate concentrations can inhibit selenate uptake due to reduced transporter selectivity, thereby decreasing selenium absorption efficiency. The translocation of selenium from roots to aerial parts largely depends on its chemical form. Selenite, which is more toxic and less mobile, is often metabolized into less toxic and less mobile organic compounds, leading to limited upward transport [34]. In contrast, selenate remains more mobile and is more efficiently translocated via the xylem. As a result, plants treated with selenate exhibit significantly higher selenium transport to shoots compared to those exposed to selenite [37].

Selenium uptake is influenced by factors such as plant species, tissue types, physiological status, and developmental stage [38]. Once absorbed, selenium is metabolized into various selenocompounds, with most plants exhibiting higher selenium concentrations in stems and leaves than in roots. For instance, seedlings often accumulate higher levels of methylselenocysteine (MeSeCys) and SeMet, while roots treated with selenate tend to accumulate Se(VI), Se(IV), and SeMet [39]. Different plant species exhibit varying capacities for selenium accumulation. Based on their selenium accumulation capacity, plants are generally classified into three categories: (1) Hyperaccumulator plants (e.g., *Stanleya* spp. and *Astragalus* spp.) can tolerate and accumulate extremely high levels of selenium, often exceeding thousands of mg/kg dry weight. These plants typically store selenium in the form of methylated selenocompounds such as MeSeCys; (2) Secondary accumulator plants (e.g., mustard, rapeseed, cauliflower, alfalfa) can accumulate moderate selenium levels (several hundred mg/kg dry weight) without exhibiting toxicity; and (3) Non-accumulator plants, including most staple crops such as wheat, maize, and rice, generally contain less than 100 mg/kg selenium (dry weight). These plants tend to sequester excess selenium in vacuoles or eliminate it via limited volatilization [12]. Hyperaccumulators, such as species of *Stanleya* and *Astragalus* (including certain tuber and bulb crops), contain the highest selenium concentrations [40]. Generally, the selenium accumulation capacity of plants follows the order: field crops (e.g., grains, legumes, and cruciferous vegetables) > leafy vegetables > seed vegetables > fruiting vegetables and tree fruits [41].

The speciation and distribution of selenium compounds vary across plant species. For example, *B. juncea* primarily accumulates MeSeCys after SeMet application; rice (*Oryza sativa*) accumulates SeMet as the dominant species, followed by MeSeCys and SeCys; garlic (*Allium sativum*) and onion (*Allium cepa*) are rich in MeSeCys, which is believed to contribute to their anticancer potential [42,43,44]. Broccoli (*Brassica oleracea* var. *italica*) contains a mixture of selenium forms, and approximately half of the accumulated selenium present as MeSeCys and SeMet [45]. The tea plant (*Camellia sinensis* (L.) O. Kuntze) is capable of absorbing soil-applied inorganic selenium (either selenate or selenite) and converting it into safer organic forms (e.g., selenoamino acids, selenoproteins, Selenopolysaccharides, and Selenium-enriched polyphenols) [46]. Similar metabolic pathways have also been reported in other crops such as wheat and tobacco [30].

**Figure 2:**
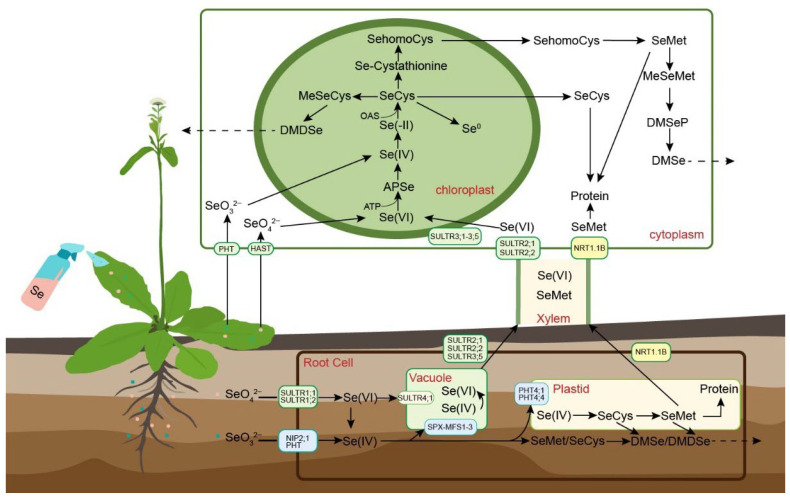
Absorption and conversion of selenate and selenite by higher plants. Abbreviations: SeO_4_^2−^, selenate; SeO_3_^2−^, selenite; Se^0^, elemental selenium; SeCys, selenocysteine; SeMet, selenomethionine; APSe, adenosine phospho selenate; OAS, *O*-acetylserine; MeSeCys, methylselenocysteine; MeSeMet, methylselenomethionine; SehomoCys, seleno-homocysteine; DMSeP, dimethylselenoproprionate; DMSe, dimethylselenide; ATP, sulphurylase. Sulfate transporters (SULTR1;1, SULTR1;2) and aquaporins (NIP2;1) are involved in uptake of selenate (Se^6+^)and selenite (Se^4+^); SULTR2;1 and SULTR2;2 help in direct transportation of Se^6+^ into shoot cells and also contribute in xylem loading of Se^6+^ in plants; NIRT, Nitrate Transporter; PHT, high-affinity phosphate transporter; HAST, high-affinity sulfate transporter; SPX-MFS1-3, SPX-Major Facilitator Superfamily Transporters 1 to 3.

Selenium metabolism in plants is illustrated as shown in Figure 2. Both selenate and selenite are taken up by plants through phosphate and sulfate transporters. Additionally, studies have demonstrated that OsNIP2;1 (also known as Lsi1), a silicon influx transporter belonging to the nodulin 26-like intrinsic protein (NIP) subfamily of aquaporins, facilitates the uptake of selenite [47]. Once inside plant cells, inorganic selenium is transported to chloroplasts, where it is assimilated via the sulfur metabolism pathway. Initially, selenate is activated to adenosine 5′-phosphoselenate (APSe) by ATP sulfurylase (APS) and subsequently reduced to selenite by APS reductase (APR) [48]. Selenite is then further reduced to hydrogen selenide (H_2_Se), either enzymatically by sulfite reductase or through a non-enzymatic reaction involving glutathione (GSH) [49]. H_2_Se reacts with O-acetylserine (OAS) under the catalysis of cysteine (Cys) synthase (also referred to as cystathionine γ-synthase) to form SeCys [50]. SeCys can follow multiple metabolic fates: it may be degraded by SeCys lyase into Se^0^ and alanine; methylated by SeCys methyltransferase to form MeSeCys; or converted through a series of enzymatic reactions into SeMet [34]. In plants, the misincorporation of free SeCys and SeMet into proteins in place of their sulfur analogs can disrupt protein structure and lead to cellular toxicity. To mitigate such toxicity, plants convert excess SeCys and SeMet into methylated forms, which are then volatilized. Methylated Se compounds such as MeSeCys and methylselenometionine (MeSeMet) can be further transformed into volatile, non-toxic gaseous selenium species—primarily dimethyl selenide (DMSe) and dimethyl diselenide (DMDSe)—which are released into the atmosphere. This volatilization pathway is especially prominent in selenium-hyperaccumulating plants, enabling them to efficiently detoxify and excrete excess selenium in gaseous form [30].

### 4.2. Types and Metabolism of Selenium-Containing Functional Factors in Plants

Selenium-containing functional compounds in plants primarily refer to biomolecules that incorporate selenium, including selenium-containing amino acids, selenoproteins, selenium polysaccharides, and selenium-enriched polyphenols [46]. Common organic selenium species found in plants include SeCys, SeMet, and their derivatives such as MeSeCys. These compounds typically exist as small peptides or are covalently bound within proteins. For example, research has demonstrated that a majority of the inorganic selenium absorbed by plant leaves is metabolized into organic selenium forms bound to soluble proteins, indicating the incorporation of selenium into proteins or peptide chains [51]. During protein synthesis, SeMet can nonspecifically replace Metin polypeptide chains, whereas SeCys, which is not directly incorporated into plant proteins, is often methylated by SeCys methyltransferase to form methylated SeCys or degraded by SeCys lyase to Se^0^, thereby minimizing its potential disruptive effects on protein structure [17]. Furthermore, selenium-enriched short peptides and polysaccharides isolated from certain fungi and plant extracts have been reported to exhibit enhanced biological activities. In plants, selenium predominantly exists in organic forms conjugated with macromolecules such as proteins and polysaccharides, which confer higher bioavailability and biological efficacy.

**Figure 3:**
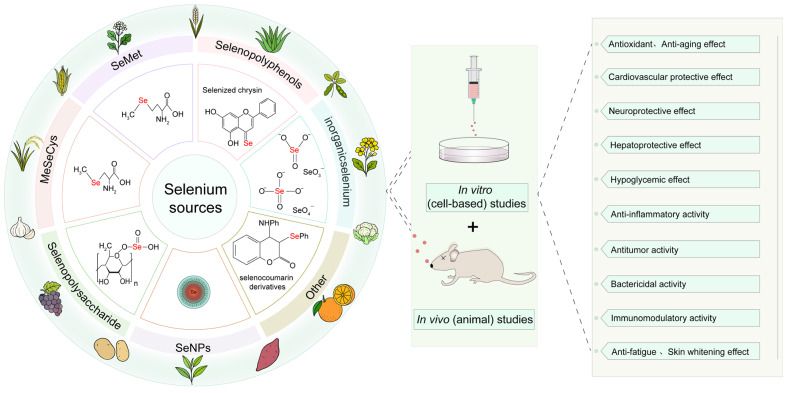
The types and bioactivities of selenium-containing compounds in plants. Abbreviations: SeO_4_^2−^ selenate; SeO_3_^2−^ selenite; SeCys selenocysteine; MeSeCys methyl-selenocysteine; SeNPs Selenium nanoparticles.

The types and bioactivities of selenium-containing compounds in plants are shown in Figure 3. These bioactive compounds exhibit a variety of beneficial effects, including antioxidant, anti-aging, cardiovascular protective, and neuroprotective properties. In addition to their direct biological activities, selenium-containing compounds from plants are used in both in vitro (cell-based) and in vivo (animal) studies to explore their potential therapeutic applications. This includes effects such as hepatoprotective, hypoglycemic, anti-inflammatory, and antimicrobial activities, as well as potential uses in anti-fatigue and skin whitening [46].

### 4.3. Absorption, Transport, Metabolism, and Utilization of Plant-Derived Selenium Compounds in the Human Body

**Figure 4:**
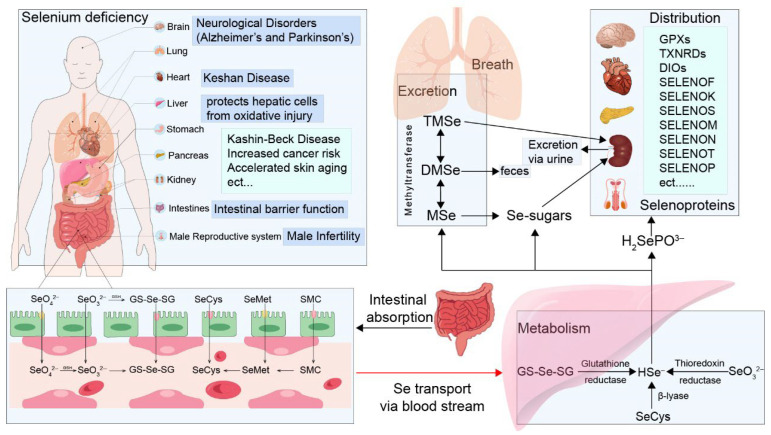
Digestion and absorption of selenium in the human body and the effects of selenium deficiency on human health. Abbreviations: SeO_4_^2−^, selenate; SeO_3_^2−^, selenite; SeCys, selenocysteine; SeMet, selenomethionine; SMC, Selenide Methylation Cycle; DMSe, dimethylselenide; TMSe, Trimethylselenonium ion; MSe, Methaneseleniol; GS–Se–SG, Selenodiglutathione; H_2_SePO^3−^, hydroselenophosphite; GSH, Glutathione; HSe^−^, Hydroselenide ion; GPXs, Glutathione Peroxidases; TXNRDs, Thioredoxin Reductases; DIOs, Iodothyronine Deiodinases; the catalytic activity of these enzymes critically depends on the presence of SeCys at their active sites.

The selenium obtained by humans from plants is predominantly in the form of organic selenium compounds, particularly SeMet and SeCys, which exhibit high bioavailability. It has been reported that the human body can absorb approximately 90% of dietary selenium [52]. The absorption, transport, and metabolic utilization of plant-derived selenium-containing compounds in the human body are illustrated in Figure 4. Selenium absorption primarily occurs in the duodenum, colon, and cecum, with different chemical species exhibiting distinct intestinal uptake mechanisms: selenite is mainly absorbed via passive diffusion across the intestinal epithelium; selenate is absorbed through a sodium-dependent cotransporter and an OH^−^ countertransport system [53]; selenium-containing amino acids such as SeMet and SeCys are taken up via sodium-dependent active transporters, specifically system B^0^ and system b^0^ + (associated with rBAT) [8]. Within intestinal epithelial cells, all selenium forms can be metabolized into H_2_Se, which serves as a central intermediate for the biosynthesis of selenoproteins, selenium-containing nucleic acids, and selenium sugars. The direct absorption efficiency of selenite is generally below 60%, as a significant proportion is converted into glutathione-bound selenodiglutathione (GS–Se–SG) within the intestinal lumen, thereby enhancing its bioavailability. A minor fraction of selenite and GS–Se–SG undergo further metabolic reduction to selenide [5]. Following absorption, selenium is predominantly transported via the bloodstream bound to plasma lipoproteins, such as low-density lipoprotein (LDL) and very low-density lipoprotein (VLDL). Upon delivery to the liver, H_2_Se is further metabolized into selenophosphate, which is utilized for the synthesis of SeCys. Under specific translational conditions, SeCys is incorporated into selenoproteins through recoding of the UGA codon in mRNA. These selenoproteins include key antioxidant enzymes such as thioredoxin reductase, glutathione peroxidase, and selenium-rich plasma proteins, among which selenoprotein P (SELENOP) plays a central role [54]. Newly synthesized SELENOP is secreted into the circulation, functioning as a selenium transport protein to deliver selenium to peripheral tissues throughout the body [55].

Different tissues in the human body exhibit variable selenium requirements. The SELENOP-mediated pathway plays a central role in delivering selenium to organs such as the kidneys, brain, and testes [56]. For instance, brain cells and renal proximal tubular epithelial cells internalize SELENOP via receptor-mediated endocytosis involving apolipoprotein E receptor 2 (apoER2) and megalin, thereby acquiring selenium [55]. Once internalized, selenium is incorporated into the active sites of essential selenoenzymes, including glutathione peroxidases, thioredoxin reductases, and iodothyronine deiodinases. These enzymes are critical for physiological processes such as antioxidant defense, thyroid hormone homeostasis, and immune regulation [57]. Overall, selenium derived from plant-based organic compounds demonstrates high absorption efficiency—approximately 90%—and is effectively utilized by the human body. In general, the bioavailability of organic selenium species surpasses that of inorganic forms.

In summary, plants absorb selenate and selenite from the soil through mechanisms similar to sulfur assimilation and convert these inorganic forms into various organic selenium compounds. These selenium species are primarily found in plants as protein-bound forms or small organic molecules. Selenium uptake and metabolism vary significantly across plant species, with cruciferous vegetables (e.g., mustard greens, cauliflower), legumes (e.g., alfalfa), and tea plants being prominent due to their efficient selenium accumulation and synthesis of organic compounds with potent antioxidant properties. Once consumed, selenium—primarily in its organic forms—is absorbed through the intestines and transported via plasma SELENOP to different organs. Selenium is then incorporated into selenoenzymes, playing a critical role in various physiological processes. Organic selenium forms are generally more bioavailable and better absorbed than inorganic forms, contributing to their greater efficiency in the human body. Ongoing research into the molecular mechanisms underlying selenium metabolism in plants will support the development of improved biofortification strategies and selenium supplementation programs. Such advancements are crucial for addressing selenium deficiencies in regions where dietary intake is insufficient, thereby enhancing public health outcomes.

## 5. Selenium Compounds and Their Biological Activity

### 5.1. Selenoproteins

Selenoproteins are proteins that contain SeCys within their amino acid sequences or incorporate selenium in other chemical forms, representing one of the primary functional forms of selenium in living organisms [58]. SeCys is often referred to as the “21st amino acid” due to its unique incorporation mechanism. Structurally analogous to Cys, SeCys differs by the substitution of the sulfhydryl (–SH) group with a more reactive and reducing selenol (–SeH) group [59]. During biosynthesis, SeCys is specifically encoded by the UGA codon—typically a stop codon—through a specialized translational recoding mechanism that facilitates its incorporation into selenoproteins, thereby conferring distinct biological activities [60].

To date, numerous selenoproteins have been identified across animals, microorganisms, and plants. In mammals, 25 selenoproteins have been characterized, with functions primarily in redox regulation, antioxidant defense, and thyroid hormone metabolism [17]. In contrast, research on plant selenoproteins remains limited, and their diversity and biological functions are not fully elucidated (Table 1). Existing evidence suggests that selenium in plants predominantly exists in organically bound forms, where selenium-containing amino acids are incorporated into peptide synthesis pathways analogous to their sulfur-containing counterparts [61]. SeMet and SeCys can compete with Met and Cys, respectively, during protein synthesis [62]. Due to the higher enzymatic efficiency and redox potential of selenoproteins compared to Cys-containing homologues, plant-derived selenoproteins exhibit enhanced antioxidant properties in humans [63]. However, many selenoproteins remain uncharacterized, including thioredoxin glutathione reductase and selenoproteins such as SelH, SelI, SelM, SelO, SelT, SelV, and SelW [64]. Among the selenoproteins with known functions, many act as redox enzymes, with SeCys at their catalytic centers, mediating diverse redox reactions [65]. Moreover, clinical studies have shown that SELENOP exerts beneficial effects in human diseases, including type 2 diabetes, Alzheimer’s disease, and cardiovascular disorders [66,67,68].

Naturally selenium-rich plants primarily include legumes, grasses, and vegetables that either grow in selenium-abundant soils or have been supplemented with exogenous selenium. Examples of such crops include soybean, wheat, tea, and maize [72,73]. These plants are capable of converting inorganic selenium into organic selenium compounds via sulfur assimilation pathways [74]. Subsequently, these selenium-containing amino acids are incorporated into polypeptide chains during protein translation, resulting in the formation of naturally selenium-enriched proteins. For example, in a study using chickpeas as the raw material, seeds were soaked in a sodium selenite solution for 6 h and then germinated under controlled conditions [75]. The selenium content in the protein extracts from germinated chickpeas increased significantly by 18-fold compared to the control group, while the total protein content increased by 38.13%. The study demonstrated that selenium supplementation not only enhanced the protein content but also increased the stability of proteins and their hydrolysates, with the selenium-enriched hydrolysates exhibiting anti-aging potential [75].

Selenium modification of natural proteins involves the stable incorporation of Se^0^ into protein molecules through covalent or coordinate bonds. This modification primarily occurs via reactive functional groups on protein side chains, such as -SH, carboxyl (–COOH), amino (-NH_2_,-NH-), and phenolic hydroxyl (–OH) groups, resulting in the formation of Se-S, Se-C, Se–N, Se–O, or Se–Se linkages. One common selenation approach involves the reaction of proteins with Na_2_SeO_3_ or selenous acid (H_2_SeO_3_) [76], where selenite or selenate directly coordinate with -NH_2_/-NH- or -OH groups to generate bonding patterns such as Se-O, Se=O, S-Se-S, and selenite bonds (-O-SeHO_2_) linkages. Zhao et al. [77] reported that selenium chelation facilitated by dry heat during protein selenation promotes the formation of–O–SeHO_2_ via interaction with amino acid –OH groups. This thermal process enhances the stability of selenite linkages, thereby improving the antioxidant capacity of the modified proteins. Furthermore, Zhang et al. [78] demonstrated that the antioxidant activity of the (Se-7S-EGCG) conjugate, produced by biofortifying selenium-enriched soybean 7S globulin (Se-7S) with epigallocatechin gallate (EGCG), was significantly higher than the Se-7S-gallic acid (Se-7S-ga) conjugate. This conjugate effectively reversed the overexpression of phosphorylated proteins within the MAPK signaling pathway, suppressed activation of associated inflammatory factors, reduced matrix metalloproteinases (MMPs) levels, and inhibited UV-B-induced apoptosis in epidermal cells.

Plant selenoproteins exhibit diverse potential functions, particularly in modulating plant responses to abiotic stresses, such as salinity, drought, and heavy metal exposure [79]. This is primarily achieved by enhancing the plant’s antioxidant defense system. Additionally, selenoproteins isolated from certain selenium-enriched plants have demonstrated pharmacological activities such as free radical scavenging, antitumor effects, and hepatoprotection [80]. These findings highlight the promising prospects of plant selenoproteins for applications in the development of functional foods, natural antioxidants, and phytopharmaceutical agents. However, due to the scarcity of selenoproteins in plants and many other organisms, their detection remains technically challenging, as they occur at extremely low abundance, exhibit considerable chemical instability, and require the complex incorporation of SeCys via UGA stop codon recoding. Proteomic identification of these proteins thus necessitates advanced methodologies, such as selenium-specific mass spectrometry and isotopic labeling, to overcome analytical limitations [81].

### 5.2. Selenopeptides

In contrast, selenopeptides can be more readily obtained using conventional LC–MS/MS approaches, and their enhanced reactivity—such as the increased nucleophilicity and redox sensitivity of Sec—combined with favorable pharmacokinetic properties, including higher solubility, improved bioavailability, and lower toxicity, makes them more suitable for the development of functional foods and pharmaceuticals [82].

Selenopeptides are bioactive oligopeptides that incorporate organic selenium species, primarily SeCys and SeMet, into their peptide backbones [83]. These peptides can be derived through several approaches, including natural bioaccumulation in selenium-rich plants, enzymatic hydrolysis, microbial fermentation, and artificial chelation methods using protein substrates [84]. Natural selenopeptides are typically isolated from selenium-accumulating plant proteins, such as those found in selenium-fortified crops like soybean, wheat, maize, tea, and wolfberry (Table 2). These are extracted using alkaline extraction followed by acid precipitation, enzymatic digestion, or microbial fermentation to yield small selenopeptides [84,85]. Peptide fractions can be selectively obtained depending on the preparation method, and selenium in peptide form demonstrates superior bioavailability compared to inorganic selenium supplements [86]. This is because selenium atoms are covalently bound to amino acid residues, facilitating efficient absorption through intestinal peptide transporters. The small molecular size and hydrophilic nature of these peptides further enhance their cellular uptake and bioactivity [87]. These peptides play an active role in intracellular antioxidative mechanisms and regulatory signaling pathways [80]. Research indicates that selenopeptides with molecular weights below 3 kDa exhibit excellent selenium-release properties in vivo, significantly elevating plasma selenium levels and glutathione peroxidase activity, thus mitigating oxidative stress-induced cellular damage. For example, Yu et al. [88] hydrolyzed selenium-enriched *Pleurotus eryngii* proteins using trypsin, separated peptide fractions (<5 kDa) by ultrafiltration, and purified them through gel filtration chromatography and reversed-phase high-performance liquid chromatography (RP-HPLC), ultimately obtaining twelve selenopeptide fragments. These isolated peptides demonstrated protective effects in Pb^2+^ induced oxidative injury in NCTC1469 cells, as evidenced by reduced nitric oxide (NO), lactate dehydrogenase (LDH), and malondialdehyde (MDA) concentrations, enhanced antioxidant enzyme activity, inhibition of apoptosis, and improved cellular viability under lead-induced stress.

In addition to naturally occurring selenopeptides, artificial bioactive selenopeptides can be synthesized via chemical synthesis or metal chelation [63]. These peptides allow precise control over amino acid sequences and selenium incorporation, converting inorganic selenium species into bioavailable organic forms, thus enhancing their biological activity and targeting specific physiological effects. Selenium atoms in these synthetic peptides often form diselenide bonds or establish selective binding interactions with specific amino acid residues, which critically affect peptide stability and biological functions [104]. Recent studies indicate that selenium mainly interacts with acidic (Glu and Asp) and basic (Lys, His, and Arg) amino acid side chains through electrostatic attractions and coordination bonding [102]. The -NH_2_/-NH- and -COOH functional groups serve as critical chelation sites for selenium binding [105]. Molecular orbital theory suggests that oxygen (O) and nitrogen (N) atoms, with their lone electron pairs, facilitate coordination bond formation [106]. Thus, the primary selenium-binding sites on peptides likely include the O atoms from -COOH and –OH groups, and N atoms from -NH_2_/-NH- groups [107,108].

Currently, two widely used methods for selenium biofortification in peptides include: (i) foliar or soil selenium fertilizer application, or seed treatment using Na_2_SeO_3_ [75]. For instance, Liu et al. [97] applied a foliar spray consisting of Na_2_SeO_3_ combined with sodium alginate to biofortify soybeans, resulting in selenium-enriched soybean peptides (SSPs) with selenium concentrations of 21.78 ± 0.17 mg/kg. Subsequent studies showed that SSP supplementation enhanced antioxidant capacity by increasing glutathione peroxidase (GSH-Px) activity and glutathione (GSH) content, improved hepatocyte viability, and suppressed hepatic stellate cell activation, effectively ameliorating liver fibrosis induced by carbon tetrachloride (CCl_4_). (ii) Chelation reactions involving peptides and inorganic selenium compounds like Na_2_SeO_3_. Ye et al. [109] successfully synthesized selenium-enriched soybean protein isolate peptides (SPIP-Se) by reacting soybean protein isolate peptides (SPIPs) with sodium selenite under controlled conditions, achieving a maximum selenium content of 46.14 mg/g. These peptides exhibited significant changes in the stretching vibrations of peptide-NH groups, confirming that-NH_2_ and-NH functional groups are essential during chelation.

Bioactive selenopeptides derived from various botanical sources offer comparable nutritional profiles to conventional peptides but exhibit superior bioactivities, including antioxidative, immunomodulatory, hepatoprotective, anticancer, antihypertensive, anti-aging, anti-inflammatory, detoxifying, and anti-fatigue properties [105]. Current research indicates that plant-derived selenopeptides are predominantly obtained from selenium-rich sources such as Cruciferae, Allium, Leguminosae, tea leaves, and cereals, and can be more efficiently produced through biofortification, germination/sprouting, enzymatic hydrolysis, or chemical selenium enrichment. The development of selenopeptides for functional foods and nutritional interventions is rapidly advancing, with promising potential in multiple domains, including antioxidant defense, metabolic syndrome management, gut–immune axis modulation, and liver and cardiovascular protection.

### 5.3. Selenopolysaccharides

#### 5.3.1. Biological Activities of Selenopolysaccharides

The presence of selenosugars, well-characterized selenium metabolites in mammalian cellular metabolism [110,111,112], implies the possibility of similar selenium-containing polysaccharides existing in plants [29]. Recent research has identified and characterized selenopolysaccharides from various botanical sources (Table 3). The incorporation of selenium into polysaccharide structures significantly enhances their biological properties, conferring improved antioxidant, antitumor, immunomodulatory, antidiabetic, neuroprotective, gut barrier-enhancing and microbiota-modulating activities [113]. Compared with polysaccharides extracted from regular tea leaves (ATPS1, ATPS2, and ATPS3), selenium-enriched polysaccharides (SeTPS1, SeTPS2, and SeTPS3) isolated from naturally selenium-rich tea have exhibited notably stronger DPPH radical scavenging abilities [114]. Cheng et al. [115] demonstrated that selenium-enriched tea polysaccharides (Se-TPS), derived from selenium-fortified tea leaves, exhibited significantly higher inhibitory efficacy against sarcoma 180 (S-180) cells compared to both conventional tea polysaccharides (TPS) and selenium-enriched yeast at equivalent doses. Moreover, selenium-enriched polysaccharides from Ziyang green tea were reported to exhibit pronounced inhibitory effects on human breast cancer MCF-7 cells [116], and Se-ZYTP from the same source showed potential in preventing and treating human osteosarcoma cells [117]. Further studies by Guo et al. [118] synthesized selenium-substituted sucrose (sucrose selenite ester) and selenium-substituted xylitol (xylitol selenite ester), which exhibited dose-dependent cytotoxic effects against human hepatocellular carcinoma SMMC-7221 cells. These selenium-polysaccharide derivatives effectively induced mitochondrial-mediated apoptosis, selectively suppressed cancer cell proliferation, and importantly, preserved the viability of normal hepatic cells. Collectively, these findings emphasize the promising potential of selenopolysaccharides as therapeutic agents in cancer prevention and treatment strategies.

#### 5.3.2. Sources and Structure of Selenopolysaccharides

Selenopolysaccharides are organic selenium-containing complexes formed through the covalent attachment (ester or ether bonds) or non-covalent interactions (hydrogen bonds, Van der Waals forces) between selenium atoms and polysaccharide molecules [134,135,136]. In plants, selenopolysaccharides are naturally synthesized with selenium biofortification methods, which include: (i) foliar spraying with sodium selenite [134], allowing direct selenium assimilation into plant foliage; (ii) cultivation in naturally selenium-rich soils, such as those in Bozhou (Anhui Province) [121], Enshi and Yichang (Hubei Province) [122], and Ankang (Shaanxi Province) [119], China; and (iii) supplementation of soil with inorganic selenium sources like Na_2_SeO_3_ or selenium-containing fertilizers [137], enabling root-mediated uptake of selenate or selenite.

Following uptake, selenium is incorporated into stable covalent or coordination complexes within plant tissues. The structures of selenopolysaccharides may include ester-type complexes (Se-O-C), Se-OH, Se-H, and Se=O groups, or bridging structures where selenium atoms are inserted between glycosidic bonds within polysaccharide chains [29]. Zhao et al. [119] successfully isolated selenopolysaccharides (SeTPS-1, SeTPS-2, and SeTPS-3) from tea leaves grown in selenium-rich soils, as confirmed by UV-visible and FT-IR spectroscopic analyses, which showed characteristic absorption peaks at 676, 799, and 1050 cm^−1^, corresponding to Se-O-C stretching vibrations, Se=O bond vibrations, and O-Se-O bonding modes, respectively. Additionally, Zhu et al. [134] identified a naturally occurring selenium-enriched tea polysaccharide (ASe-TPS1) that exhibited distinct FT-IR absorption bands at 611, 669, and 1080 cm^−1^, attributable to Se-O-C, Se-H, and O-Se-O stretching vibrations, respectively. Despite these promising findings, the relatively low natural abundance of selenopolysaccharides remains a limitation, restricting their large-scale extraction, further mechanistic studies, and industrial applications.

However, elevated concentrations of selenopolysaccharides can be achieved in plants via targeted biofortification strategies, including foliar spraying of selenium solutions onto plant leaves or the application of selenium fertilizer to the soil [138]. Additionally, polysaccharides can be chemically modified through controlled selenization to yield synthetic Se-polysaccharide derivatives [139]. Integration of inorganic selenium compounds with naturally derived polysaccharides effectively mitigates the inherent limitations associated with naturally sourced selenopolysaccharides, such as low availability and insufficient selenium content. Importantly, the bioactivities of these selenized polysaccharides often substantially exceed those of either selenium or polysaccharides individually [104].

Several established methodologies for polysaccharide selenization have been developed: (i) Microwave-assisted synthesis using HNO_3_-Na_2_SeO_3_/H_2_SeO_3_ system with BaCl_2_ as a catalyst: Yuan et al. [131] synthesized selenium-modified sweet potato polysaccharides (Se-SWP), achieving selenium contents as high as 12.74 mg/g. This product demonstrated significant antioxidant activity, potent tumor proliferation inhibition (>50%), and improved immunomodulation in diabetic rat models; (ii) Microwave-assisted synthesis utilizing selenium oxychloride (SOC): Zhu et al. [128] reported synthesizing selenized Artemisia sphaerocephala polysaccharides (SeASP) with selenium content (22,400 μg/g), showing significant antiproliferative activity against three tumor cell lines; (iii) Selenization with CH_3_COOH-Na_2_SeO_3_/H_2_SeO_3_ systems: Gao et al. [129] comparatively investigated four distinct methods-HNO_3_-Na_2_SeO_3_, CH_3_COOH-Na_2_SeO_3_, CH_3_COOH-H_2_SeO_3_, and SeOCl_2_-for chemically modifying garlic polysaccharides (GPS). Among these, the HNO_3_-Na_2_SeO_3_ system yielded the highest selenium content (29.4 mg/g), followed by CH_3_COOH-H_2_SeO_3_ (26.3 mg/g), CH_3_COOH-Na_2_SeO_3_ (10.5 mg/g), and SeOCl_2_ (9.2 mg/g). Selenization substantially improved the immunomodulatory activities of GPS, surpassing the biological effects of unmodified polysaccharides; (iv) AlCl_3_-Na_2_SeO_3_ microwave-assisted approach: Zhao et al. [140] utilized selenopolysaccharides from *Platycarya strobilacea* infructescence polysaccharides under microwave irradiation, efficiently synthesizing selenopolysaccharides with a selenium content of 3.58 mg/g. Compared to nitric acid catalysis, this method significantly reduced reaction time and minimized acidic waste generation; (v) Introduction of selenium-containing functional groups via covalent and non-covalent interactions: Chen et al. [141] introduced synthesized selenium diacetate into chitosan (CS), forming O-selenium-diacetyl-chitosan (OSAC) and chitosan-ammonium selenium diacetate (CASA) with selenium contents reaching 15,720 ± 475 and 26,363 ± 698 μg/g, respectively. Compared to native CS, OSAC and CASA exhibited significantly enhanced DPPH and ABTS radical scavenging activities and demonstrated pronounced anticancer efficacy against HepG2 hepatocellular carcinoma cells. These advances in selenium-polysaccharide synthesis and modification offer promising avenues for the development of functional foods, natural antioxidants, and phytopharmaceutical agents.

### 5.4. Selenium-Enriched Polyphenols

Polyphenolic compounds, including phenolic acids, flavonoids, stilbenes, and lignans, constitute a diverse class of bioactive natural secondary metabolites characterized by –OH groups attached to aromatic frameworks. As major exogenous antioxidants, polyphenols exhibit multifaceted biological activities—antioxidant, anti-inflammatory, antimicrobial, anticancer, neuroprotective, and cardioprotective effects—primarily by modulating critical cell-signaling pathways [142] (Table 4). Sentkowska and Pyrzynska [143] identified a synergistic antioxidant interaction between selenium and tea polyphenols, demonstrating a substantial increase in polyphenolic content in selenium-enriched teas. Selenium-enriched polyphenols, formed by covalent integration of selenium atoms into polyphenol structures or by forming polyphenol-selenium conjugates, not only significantly enhance the antioxidant stability of polyphenols but also leverage the biological advantages inherent to selenium. Lin et al. [144] synthesized six novel selenium-containing polyphenolic esters via a Mitsunobu reaction, coupling polyphenolic acids with 2-phenylselenoethanol. Among these, three derivatives exhibited potent antioxidant and anti-inflammatory activities, markedly inhibiting 5-lipoxygenase with efficacy comparable or superior to caffeic acid phenethyl ester (CAPE). Structurally, selenium atoms in Selenium-enriched polyphenols preferentially bind to electron-deficient sites, such as positions C6 and C8 of the flavonoid/catechin A-ring, the oxidized o-diphenol structure (quinone ring) in the flavonoid B-ring, and the aromatic rings of phenolic acids (e.g., gallic acid) [145,146]. Polyhydroxylated flavonoids, including gallic acid [147], EGCG [148], quercetin [149], and dihydromyricetin [150], are particularly suitable for selenium conjugation. Zhang et al. [151] synthesized selenium-vitamin P complexes (SEVP) by reacting flavonoid derivative vitamin P with SeOCl_2_. Structural analysis via ^1^ H NMR revealed the formation of O-Se-O bonds due to the modification of –OH groups. The resulting SEVP complex exhibited efficient binding to human serum albumin without significantly altering the secondary structure of bovine serum albumin.

Further developments include the synthesis of selenium-modified nanoparticles. Zhou et al. [148] synthesized stable SM-EGCG-SeNPs nanoparticles by reacting corn starch gel (30% oxidation degree) and EGCG-stabilized selenium nanoparticles (SeNPs) in an ascorbic acid–H_2_SeO_3_ reduction system. Spectroscopic analyses (UV-vis, FT-IR, XPS, XRD) confirmed intermolecular Se-O interactions between starch, EGCG, and SeNPs, resulting in enhanced nanoparticle stability compared to starch-only SeNPs. Additionally, Fiorito et al. [153] synthesized the first selenium-substituted phenylpropene compound, selenoauraptene, from auraptene, a natural bioactive compound abundant in citrus fruits and pomegranate, via Newman–Kwart rearrangement. This compound incorporated selenium functionalities at the 7th position of the umbelliferone moiety. Furthermore, Zhou et al. [147] synthesized SC@Se/GA nanoparticles by reacting gallic acid and sodium caseinate, naturally abundant in plants and fruits, with an ascorbic acid–Na_2_SeO_3_ system. FTIR characterization verified complete reduction of Se^4+^ to Se^0^, yielding nanoparticles with superior storage stability and dispersibility for potential biomedical and nutraceutical applications.

Most studies on Selenium-enriched polyphenols have focused on chemical synthesis, but plant extract-mediated Se-polyphenol systems have demonstrated promising gastrointestinal bioavailability and enhanced biological effects. Zhang et al. [151] reported that SEVP effectively inhibited HT29 colon cancer cell proliferation by arresting DNA synthesis during the S phase of the cell cycle. Similarly, Zhou et al. [148] constructed SM-EGCG-SeNPs, which exhibited superior colloidal stability, heightened bioactivity, and potent cytotoxicity, inducing apoptosis in cancer cells through activation of multiple caspases and overproduction of reactive oxygen species (ROS). Furthermore, Zhou et al. [147] synthesized SC@Se/GA nanoparticles, which displayed robust anti-inflammatory and antioxidant properties and effectively released selenium species with significant nephroprotective potential.

### 5.5. Nano-Selenium

Nano-selenium (Nano-Se) refers to selenium-based nanoparticles, typically ranging from 1 to 1000 nm in size (commonly 5–350 nm), composed of Se^0^ or its reduced derivatives (e.g., from SeO_2_) [154]. Nano-Se uniquely integrates the essential biological activity of selenium with the physicochemical advantages of nanomaterials, particularly a high surface area-to-volume ratio, enhanced reactivity, and tunable bioavailability [155]. These properties make nano-Se a promising candidate in various fields such as food fortification, nutraceuticals, and targeted biomedical delivery systems [156].

Two primary approaches for synthesizing nano-Se are chemical reduction and biogenic synthesis [154]. Alternative physical approaches for nano-Se synthesis, including pulsed laser ablation and ultrasound-assisted methods, are falling out of favor owing to limited cost-effectiveness and significant environmental, biosafety, and toxicity concerns [157]. In the chemical method, selenium precursors (e.g., sodium selenite or selenic acid) are reduced using agents such as potassium borohydride or ascorbic acid, often in the presence of stabilizing polymers like polyvinyl alcohol (PVA) or polyvinylpyrrolidone (PVP) [158]. These methods offer precise control over particle size and dispersity, enabling the production of monodisperse and size-tunable SeNPs (Selenium Nanoparticles). However, the reliance on strong reductants and elevated temperature or pressure conditions introduces potential drawbacks, including chemical residue contamination and environmental concerns [159]. For example, El-Ghazaly et al. [160] synthesized ~13 nm spherical SeNPs using a PVP/KBH_4_ reduction system, while Tran et al. [161] produced ~70 nm particles by reducing sodium selenate with ascorbic acid. To mitigate cytotoxicity, biocompatible coatings, such as polysaccharides or proteins, can be incorporated, enhancing the stability and compatibility of chemically prepared SeNPs.

In comparison, biological synthesis methods, which utilize microbial and plant-derived systems, offer a more sustainable alternative by avoiding harsh chemical reagents and energy-intensive conditions [159]. Microbial systems leverage the metabolic pathways of organisms such as bacteria, fungi, and yeasts to convert selenium precursors into SeNPs. Specific strains, including *Lactobacillus*, *Pseudomonas*, and *Streptomyces*, have demonstrated the capacity to reduce toxic selenium salts into biologically safe selenium nanoparticles [162]. For instance, *Lactobacillus paralimentarius* JZ07 exhibits a strong capacity for selenium biotransformation, predominantly accumulating Se^0^by reducing sodium selenite into amorphous selenium nanospheres (150–300 nm in diameter) that are deposited extracellularly [163]. Under selenium stress, notable morphological alterations were observed in JZ07 cells. Similarly, the yeast *Yarrowia lipolytica* is capable of synthesizing SeNPs (~110 nm) enveloped in protein- and lipid-rich layers [164]. While biologically synthesized SeNPs generally exhibit superior biocompatibility, their particle size distribution tends to be broader (ranging from 10 to 200 nm), and the production process requires strain-specific cultivation and bioprocessing expertise [164].

Plant-based green synthesis has emerged as a cost-effective, eco-friendly strategy for producing SeNPs. Plant bioactive compounds such as proteins, enzymes, polysaccharides, terpenoids, flavonoids, tannins, and phenolic acids serve as reducing agents, converting selenium precursors (e.g., Na_2_SeO_3_) into Se^0^ [158], while also acting as capping agents to stabilize the nanoparticles. These reactions typically proceed under mild conditions, often at room temperature and without the need for toxic chemical additives or high energy input, making the process cost-effective, scalable, and eco-friendly [157]. Numerous plant extracts have been used to synthesize SeNPs, including neem (*Azadirachta indica*) leaf extract, which has shown potent antibacterial activity against both Gram-positive and Gram-negative pathogens [165]. Other plants used for nano-Se synthesis include okra, olive pomace, herbs, walnut leaves, moringa oleifera, tea, garlic, and many others [157,158,159,166]. The resulting SeNPs are generally encapsulated by natural biomolecules, imparting a negatively charged surface, excellent colloidal stability, and low cytotoxicity [157]. These nanoparticles are biocompatible, safe for parenteral administration, and unlikely to cause secondary environmental pollution [167]. In recent years, plant-mediated nano-Se synthesis has been widely endorsed as a core technique in green nanotechnology, holding significant promise for applications in food fortification, antimicrobial materials, and biomedical delivery systems [168].

SeNPs, with their multifunctional properties, have emerged as a platform with broad biomedical applications, including antioxidant defense, antimicrobial action, anticancer therapy, and drug delivery [157]. SeNPs exhibit a dual mechanism of cytoprotection—direct scavenging of ROS and upregulation of endogenous antioxidant systems—making them a new-generation antioxidant with enhanced biological relevance [164]. Studies have shown that SeNPs can restore the activity of key antioxidants such as vitamin C and coenzyme Q10 [169,170]. In plant-mediated synthesis, the presence of polyphenols further enhances the antioxidant potential of SeNPs, amplifying ROS neutralization and mitigating oxidative cellular damage [171].

In terms of antimicrobial activity, SeNPs demonstrate broad-spectrum efficacy against a variety of bacterial and fungal pathogens. Their mode of action is similar to that of metal-based nanoparticles, involving disruption of microbial membranes, leakage of cytoplasmic contents, and induction of oxidative stress, leading to microbial death [172]. In vitro studies have confirmed the inhibitory activity of plant-derived SeNPs against both Gram-positive (*S. aureus*, *B. subtilis*) and Gram-negative (*E. coli*, *P. aeruginosa*) strains [159]. Borna et al. [173] synthesized SeNPs using *Pelargonium* leaf extract under microwave irradiation and reported significant antibacterial effects against *E. coli* and *S. aureus*, as well as antifungal activity against *Colletotrichum* spp. and *Penicillium digitatum*.

In addition to their antimicrobial effects, SeNPs also exhibit compelling anticancer properties across multiple tumor models. For example, in methicillin-resistant *S. aureus* (MRSA) models, SeNPs not only directly inhibit bacterial growth but also enhance lincomycin efficacy by promoting bacterial protein degradation, offering a promising approach to combat antibiotic resistance [172]. Their mechanism involves intracellular ROS generation and mitochondrial dysfunction, leading to activation of apoptotic signaling pathways, induction of programmed cell death, and inhibition of cancer cell proliferation, with minimal cytotoxicity to healthy cells [174]. Kasi et al. [175] reported that SeNPs synthesized from aqueous garlic extract effectively suppressed cytotoxic responses in Vero cells. Additionally, SeNPs also function as versatile nanocarriers in targeted drug delivery. Surface modification with tumor-targeting ligands such as folic acid or saccharides enables selective recognition and binding to tumor-associated receptors. For instance, Vennila et al. [176] synthesized folate-conjugated SeNPs using *Sargassum wightii* extract (SAG-sh-SeNPs) to deliver S-allyl glutathione to HepG2 liver cancer cells, resulting in cell cycle arrest and apoptosis induction.

Beyond therapeutic applications, SeNPs have attracted increasing attention in biosensing applications. Mostafavi et al. [177] provided a comprehensive overview of SeNP-based biosensors capable of detecting biologically relevant analytes such as hydrogen peroxide, glucose, and heavy metal ions. The excellent sensitivity, selectivity, and multifunctionality of SeNP-based platforms make them highly suitable for next-generation biomedical diagnostics and clinical monitoring systems, opening new frontiers in precision medicine and point-of-care technologies.

Beyond the commonly studied selenium forms, a variety of novel selenium-containing compounds, such as diselenides, selenoesters, methylseleninic acid, 1,2-benzisoselenazole-3(2H)-1, selenenyl derivatives, and selenols, have attracted increasing attention for their promising biomedical applications. Gao et al. [178] developed a selenium-mediated nanogel system via a multicomponent reaction (MCR) involving low-molecular-weight polyethyleneimine (LMW PEI), γ-selenobutyrolactone (γ-SBL), and poly(ethylene glycol) methacrylate (PEGMA). The resulting nanogel, A(1.8)Se(3)O(0.5)/siPD-L1, was stabilized through dynamic diselenide linkages and exhibited potent antitumor activity by suppressing autophagy and blocking immune escape via lysosomal alkalinization—highlighting a novel strategy for immunotherapy and gene delivery. Cheng et al. [104] further reviewed cyclic organoselenium compounds synthesized via transition-metal-catalyzed cross-coupling reactions of arylboronic acids or aryl halides with selenium sources. These compounds demonstrated potent antioxidant, anticancer, and radioprotective activities, expanding the functional diversity of selenium-based small molecules. A particularly notable compound, Selol, a selenium-containing triglyceride mixture, was synthesized by hydroxylating unsaturated fatty acids from sunflower oil using potassium permanganate, followed by a reaction with selenious acid in dioxane under the catalytic action of amorphous selenium [179]. Selol contains Se^4+^, allowing it to exert antioxidant and cytoprotective effects while mitigating the inherent toxicity of inorganic selenium ions. Selol has demonstrated significant anticancer activity across multiple cancer cell lines, including the modulation of redox homeostasis in androgen-dependent prostate cancer (LNCaP) and the protection of neuronal PC12 cells from sodium nitroprusside (SNP)-induced oxidative stress and apoptosis. These findings suggest that Selol not only serves as a potential pro-apoptotic agent in tumor therapy but also as a safe selenium supplement for individuals with selenium deficiency [180].

## 6. Applications and Prospects of Selenium Compounds

A growing body of research highlights that selenium supplementation should not merely focus on dosage but should consider the complex interplay between selenium speciation and individual physiological status. This nuance must be addressed within the narrow “selenium intake safety margin” to avoid toxicity while optimizing its benefits. Enhancing selenium bioavailability is key to increasing systemic selenium levels without exceeding toxic thresholds [181]. However, clinical data on the threshold limits of different selenium species remain limited [10]. Organic selenium compounds are generally more bioavailable and exhibit lower toxicity than their inorganic counterparts [5]. In this context, selenium-enriched foods offer a cost-effective and sustainable approach to dietary selenium augmentation [182]. Common selenium-rich foods include Brazil nuts, selenium-enriched broccoli, green tea, spirulina, and fungi. In addition, inorganic selenium can be bio-converted into organic forms through plant, animal, or microbial pathways to produce value-added selenium-fortified products, such as selenium drinks, soybean protein selenium, selenium tea polysaccharides, selenium-enriched garlic powder, gold flower selenium protein, wild rapeseed selenium capsules, and organic selenium lycopene soft capsules, etc. [183,184]. Advances in understanding intestinal selenium absorption and the molecular mechanisms of selenoproteins hold promise for improving selenium bioavailability and facilitating more personalized nutritional interventions for health maintenance and disease prevention.

### 6.1. Selenium-Based Strategies in Drug Development and Therapeutic Applications

Targeted Anticancer Therapies and Immunomodulation: Organic and nano-formulated selenium compounds have demonstrated remarkable potential in oncology. Functionalizing SeNPs with targeting ligands, such as peptides or antibodies, enables selective drug delivery to tumor cells. For instance, GE11-peptide-conjugated SeNPs (GE11-ori-SeNPs) loaded with oridonin—a bioactive compound from traditional Chinese medicine—successfully targeted EGFR-overexpressing cancer cells, enhancing cellular uptake, inhibiting tumor growth, and reducing off-target toxicity. The anticancer efficacy of these formulations is attributed to ROS generation, suppression of PI3K/Akt and Ras/Raf/MEK/ERK signaling pathways, and enhanced anti-tumor immune responses, including the upregulation of IL-2 and TNF-α and the inhibition of angiogenesis [185]. Selenium compounds are recognized for their antioxidant and immunoregulatory functions. Selenium-dependent enzymes such as glutathione peroxidase and selenopeptides activate the Nrf2 signaling cascade and inhibit NF-κB-mediated inflammatory pathways, thereby mitigating chronic inflammation and offering organ-protective effects, particularly in hepatic tissues [186]. Moreover, selenium compounds have shown notable neuroprotective effects, particularly in mitigating oxidative stress, neuroinflammation, and mitochondrial dysfunction, thereby offering therapeutic potential for neurodegenerative disorders such as Alzheimer’s disease, Parkinson’s disease, and ALS. Mechanistically, selenium influences infertility by modulating antioxidant defenses in reproductive tissues, regulating testosterone biosynthesis, and improving sperm quality; it supports cardiovascular health through the inhibition of LDL oxidation, endothelial protection, and anti-inflammatory signaling; and in type 2 diabetes, it contributes to glycemic control by enhancing insulin sensitivity and protecting pancreatic β-cells from oxidative damage [10]. Additionally, selenium and its compounds—particularly selenoamino acids, selenoproteins, and selenium nanoparticles—are under active development or application for the treatment of heavy metal–related diseases, functioning through chelation, redox buffering, and the restoration of metal-induced enzymatic inhibition [184].

Stimuli-Responsive Drug Delivery Platforms: The reversible nature of diselenide bonds has led to the design of stimuli-responsive polymeric materials for controlled drug release. Zhang et al. [187] synthesized diselenide-containing polymers derived from γ-selenobutyrolactone, which exhibit light or reduction-triggered cleavage, enabling smart release and potential use in self-healing biomaterials. Selenium-infused hydrogels and wound dressings—such as SeNP-coated bandages and fabrics—have shown great promise due to their biocompatibility, moisture retention, antibacterial efficacy, and adhesion to wound surfaces, making them ideal candidates for localized and sustained therapeutic delivery in clinical wound care [188]. In the context of HIV therapy, selenium-based compounds have demonstrated notable anti-HIV activity, highlighting the immunomodulatory and antimicrobial potential of selenium in combating viral and bacterial infections. Addressing the challenge of drug resistance or suboptimal efficacy in infected individuals, Faith et al. reported that a combination of GP and C60 modified with chalcogens—particularly O, S, and Se—can facilitate the targeted delivery of zidovudine [189]. Beyond HIV, selenium has shown potential roles against viral mutations and diseases caused by pathogens such as SARS-CoV-2 and influenza A virus, highlighting its importance in antiviral strategies and in advancing the understanding of the genetic and immunological aspects of viral infections [190].

### 6.2. Applications of Selenium in Food and Nutrition: Biofortification and Health Implications

Selenium is a vital trace element required for maintaining human health. In China, approximately two-thirds of the national territory—affecting nearly 700 million individuals—falls within selenium-deficient regions. Several epidemiological investigations have indicated a strong association between low selenium (Se) status in certain regions of China and an increased prevalence of thyroid disorders. For instance, populations residing in areas of Shaanxi Province characterized by habitually low dietary Se intake exhibit a significantly higher incidence of thyroid diseases compared with neighboring control regions. Prospective cohort studies further reveal that individuals with Se deficiency have a greater risk of developing Hashimoto’s thyroiditis and display elevated titers of anti-thyroid peroxidase antibodies (TPOAb), underscoring a potential link between regional Se insufficiency and thyroid autoimmunity. Moreover, multiple clinical trials and meta-analyses have demonstrated that appropriate Se supplementation—particularly among Se-deficient individuals or those with autoimmune thyroid disease—can reduce circulating levels of TPOAb and anti-thyroglobulin antibodies (TGAb), and in some cases ameliorate clinical symptoms or improve ultrasonographic features [191,192]. These findings reinforce the public health importance of addressing Se–thyroid interactions in Se-scarce regions. Notably, thyroid disorders frequently coexist with gastrointestinal conditions, and modulation of the gut microbiota can influence Se supplement absorption, thereby impacting the activity of Se-dependent enzymes and the metabolism of thyroid hormones [193]. Therefore, Se supplementation strategies should be individualized according to baseline Se nutritional status, adhere to clinical guidelines, and account for the dynamic interplay with gut microbial ecology and its implications for long-term clinical outcomes, as well as the impact on improving quality of life [194]. This widespread deficiency has intensified efforts to develop selenium-enriched functional foods and dietary supplements, particularly focusing on bioavailable and low-toxicity selenium forms.

Organic selenium compounds—such as selenium-enriched yeast, L-SeMet, and selenopolysaccharides—are considered superior due to their efficient metabolic conversion into SeCys and SeMet, which are readily incorporated into selenoproteins. Comparative studies in ruminants have demonstrated that organic selenium significantly enhances tissue selenium deposition and antioxidant enzyme activities, outperforming inorganic forms in both efficacy and safety [35]. Among selenium-functionalized biomaterials, selenate polysaccharides (e.g., selenized carrageenan) have shown great potential as nutritional therapeutics. These compounds offer non-toxic, highly bioavailable, and anti-inflammatory/anticancer properties, making them attractive for the dietary management of conditions such as inflammatory bowel disease (IBD) [195]. Conversely, inorganic selenium, while inexpensive and abundant, poses concerns due to low bioavailability and a narrow therapeutic window, and is primarily utilized as a precursor in selenium-enriched food processing or as a feed additive rather than as a direct supplement. Expanding the development of selenium-fortified foods and promoting agronomic biofortification strategies is essential to combat selenium deficiency on a population scale. Special attention should be given to edible crops capable of moderate selenium accumulation, following a general trend in selenium uptake efficiency: Brassica vegetables > legumes > cereal grains [30].

### 6.3. Applications of Selenium in the Cosmetics and Skincare Industry

(i) Antioxidant and Anti-Aging Effects: As an essential trace element involved in the body’s antioxidant defense system, selenium is widely recognized as one of the “anticancer kings” and has shown beneficial effects on skin aging [186]. Studies have indicated that adequate selenium levels enhance the body’s capacity to scavenge free radicals, maintain immune homeostasis, and thereby delay cellular aging [196]. In skincare applications, selenium has been used to protect the skin against UV radiation and environmental oxidative stress, helping to preserve the structure and elasticity of collagen. (ii) Whitening and Skin Repair Functions: Experimental studies have revealed that selenium-containing compounds can inhibit melanin production and promote skin regeneration. Wei et al. [197] investigated selenium-enriched mung bean fermentation broth (Se-MBFB), which is rich in polyphenols, peptides, and γ-aminobutyric acid (GABA), and demonstrated significant free radical scavenging and tyrosinase-inhibitory activities. These effects reduced melanin synthesis and upregulated aquaporin-3 (AQP3) expression, contributing to moisturization in human dermal fibroblasts (HDFs). Yang et al. [198] applied Bombyx batryticatus peptide-templated nano-se particles (BBPP-SeNPs) to the skin and found that they exhibited potent free radical scavenging capacity and tyrosinase inhibition, significantly improving UV-induced hyperpigmentation. In vivo testing further confirmed that selenium dressings protected wounds from bacterial infection within 5 days. Compared with traditional skin whitening agents, these selenium-based materials offer improved photostability, oxidative resistance, and excellent biocompatibility, highlighting their strong potential in skin whitening and repair. (iii) Regulation of inflammation and treatment of alopecia: More than 50% of the population is affected by hair loss, with androgenetic alopecia (AGA) being the most prevalent type. Jing et al. developed a bio-composite microneedle system composed of selenium nanozymes encapsulated within hypoxia-induced extracellular vesicles (Se-HEVs-AMN). The microneedles were modified with Astragalus polysaccharides (APS) and loaded with Se-containing nanozymes embedded in hypoxia-induced EVs. When applied to the dorsal skin of AGA-model mice, this system modulated local inflammation, promoted angiogenesis, scavenged reactive ROS, and counteracted androgen activity, thereby increasing hair density and enlarging hair follicle diameter [199]. Tahoora et al. [200] reported that serum selenium levels in patients with genital warts were significantly lower than those in healthy controls, suggesting a potential association between diminished immune competence and heightened inflammatory status. In parallel, Elmaaty et al. [201] demonstrated that embedding selenium nanoparticles into textiles conferred potent antibacterial and antiviral activity—including efficacy against SARS-CoV-2—while eliciting minimal skin irritation, thereby highlighting their substantial potential for preventing skin infections. In the future, selenium-rich peptide extracts and nano-se carriers are expected to become novel skincare ingredients with combined antioxidant, whitening, and anti-inflammatory properties.

### 6.4. Medical Consumables and Biomaterials

(i) Antimicrobial Coatings and Implantable Devices: Selenium-based nanomaterials have gained attention as antimicrobial surface coatings for infection-prone medical devices. In particular, nano-se coatings on orthopedic implants have demonstrated strong efficacy in preventing colonization by multidrug-resistant pathogens, such as methicillin-resistant *Staphylococcus aureus* (MRSA) [202]. Both in vitro assays and animal models confirm that selenium coatings can substantially reduce microbial load, providing an antibacterial barrier that supports tissue integration and complex wound healing in clinical settings. (ii) Selenium-Infused Dressings for Wound Management: Selenium-functionalized materials have shown significant promise in advanced wound care applications. For instance, Tran et al. [188] reported the fabrication of antibacterial dressings by covalently grafting organic selenium compounds onto cotton textiles. In murine wound models, these selenium-coated gauzes provided complete protection against bacterial infection, maintaining antimicrobial efficacy even after repeated washing—no bacterial presence was detected on the dressing or wound area up to five days post-application. Moreover, selenium-containing polymeric dressings and nano-se hydrogels offer additional benefits, including moisture retention, enhanced tissue regeneration, and sustained antimicrobial release. (iii) Next-Generation Functional Biomaterials: Selenium-containing polymers and nanostructures are being explored for their integration into intelligent biomedical systems [177]. Diselenide-crosslinked polymers, owing to their redox-responsive reversible bonding, are particularly suited for stimuli-sensitive drug delivery, self-healing coatings, and environment-adaptive materials [188]. Thanks to their biocompatibility and redox versatility, selenium-based systems have also been applied in the design of biosensors and bioelectronic devices, where they function as sensing elements or signal transducers [202]. Collectively, selenium-enriched biomaterials present a highly versatile platform for the development of multifunctional wound dressings, antibacterial coatings, responsive drug delivery systems, and next-generation biosensing technologies, bridging material innovation with clinical and diagnostic advancements.

## 7. Conclusions

Selenium deficiency represents a widespread global health concern, closely linked to the onset and progression of numerous human diseases. As the primary dietary form of selenium for humans and livestock, plant-derived organic selenium plays a central role in determining selenium’s bioavailability and physiological efficacy. The speciation, accumulation patterns, and chemical forms of selenium in plants critically influence its nutritional value and biological functionality. Advancing our understanding of plant-based selenium compounds, including their diversity, enrichment mechanisms, chemical states, and concentration levels, is essential for unlocking their therapeutic potential. However, significant challenges remain in evaluating their bioactivity through in vitro assays, animal models, and especially clinical validation. Overcoming these barriers will be pivotal for translating selenium biology into real-world applications. The consolidated research in this review provides a compelling framework for the development of selenium-enriched functional foods, anticancer therapeutics, dermaceuticals, and sustainable bio-based materials. Collectively, these innovations hold immense promise for mitigating selenium deficiency-related health conditions and for improving the quality of life on a global scale.

## Figures and Tables

**Table 1:** Sources and biological activities of selenoproteins from various plant species.

Plant Source	Se Source	Selenium Speciation	Se Content (μg/kg)	Biological Effects	Reference
Soybean	Artificial se-enriched	SeMet; MeSeCys; SeCys	2882; 2035; 4301	Antioxidant activity	[69]
Soy	Natural	/	339.25	Antioxidant activity	[70]
Rice	Natural	/	22,010 ± 340 (Water-soluble protein); 8260 ± 400 (Alkali-soluble protein); 1670 ± 70 (Salt-soluble protein); 73 ± 130 (Alcohol-soluble protein)	Liver protection; Immunomodulatory effects; Anti-aging activity; Antioxidant activity	[71]

**Table 2:** Sources and biological activities of selenopeptides in various plants.

Plant Source	Se Source	Peptide Sequences	Selenium Speciation	Se Content in Se-Peptides(µg/g)	Biological Effects	Reference
*Cardamine violifolia*	Natural	GRVGSSSeC; GRAGGSYSeM; GHPNFKLNSeCGG; GTKSSeCKA; ASSNARDSeMI; TAGGSeCYIPI; MeSeCALQ	SeCys; SeMet; MeSeCys	505 ± 22~2970 ± 16 (CPR1-15);	Antioxidant activity	[89]
	Natural	YLPGSeMV; FSeCLVEST; VHTSeCPISeCTS; LLTMeSeCPA; SVIATISeMVP; SSeCSeCSPTP; KKSeCSL; CPQSMeSeK; NSeCVASPL; NLIVNSeMKN	SeMet; SeCys; MeSeCys	2450 ± 80 (SPE); 914 ± 18 (SPE1); 1841 ± 24 (SPE2); 1987 ± 37 (SPE3); 3104 ± 55 (SPE4)	Antioxidant activity; anti-fatigue activity	[90]
	Artificial se-enriched	/	/	/	Protective effects against brain aging; alleviation of neuroinflammation; antioxidant activity	[91]
Rice	Natural	SeMet-Pro-Ser; Met-MeSeCys-Glu; SeMet-MeSeCys-Glu	SeMet; MeSeCys; MeSeCys-Glu	3.62 (SPHs); 0.875 ± 0.044~4.154 ± 0.049 (SPHs 1-4)	Immunomodulatory effects; anti-inflammatory activity; reduction of Pb^2+^-induced cytotoxicity	[92]
Rice	Natural	TSeMMM; SeMDPGQQ	SeMet	8.95 ± 0.02~13.61 ± 0.01 (SPHA/N/T/P/F7)	Immunomodulatory effects; anti-inflammatory activity; antioxidant activity; neuroprotective effect	[93,94]
Soybean	Natural	SSeCAHK	SeCys	110.4 (SSP)	Anti-fatigue activity; inhibition of lipid peroxidation	[95]
Soybean	Natural	/	SeCys; SeMet	90.03 ± 3.23	Anti-fatigue activity	[96]
Soybean	Artificial se-enriched	SeMVVSeC; SSeCRDCV; FI/LFSeCF; SeCI/LSSeC	SeCys	21.78 ± 0.17 (SSPs)	Antioxidant activity; liver protection	[97]
Corn	Natural	SeMet-MeSe Cys-Glu; Met-MeSeCysGlu; MeSeCys-Glu-Asp; Ile-MeSeCys-Glu	SeMet; SeEt; γ-GluMeSeCys; MeSeCys; SeCys_2_	6.05	Alcohol detoxification effect	[98]
Corn	Artificial se-enriched	FSeC; WSeMQE; WSeMKE; SeCYE; FMSeCM; SeCCAMSeC; FMSeCVQ; FMSeCVK	SeCys; SeMet; MeSeCys	32.37 (SeCPs)	Antioxidant activity; inhibition of lipid peroxidation; liver protection	[99]
Corn	Artificial se-enriched	FLPPVTS; IGPRLPWPE; IIGGA; LLPPY	/	428.95 (LLPPY)	Antioxidant activity	[100]
Pea	Artificial se-enriched	PPKIYP	Se^4+^ forms covalent bonds through coordination with-NH_2_ or -COOH groups.	29.47 (PPKIYP)	Antioxidant activity	[101]
*Moringa oleifera* *Seeds*	Natural	FLSeML; mLSe- MAAL; mmLASeMMVL; mSeMLLAA; mLSeMAL	SeMet; SeCys	13.772 (F1-3); 2.253 (protein hydrolysate)	Antioxidant activity	[102]
Tea	Natural	LQPSLGFP; mAETGEIKGHY	/	2.82 (Se-TAP); 2.25 (Se-TAPep 1)	Antihypertensive activity (ACE inhibitory activity)	[103]
chickpea	Artificial se-enriched	/	/	8.84 ± 1.65 (Protein extract)	Anti-aging effect	[75]

**Table 3:** Sources and biological activity of selenopolysaccharides in plants.

Plant Source	SePS	Se Source	Se Content in SePS(µg/g)	Se Bond	Biological Effects	Reference
Tea	ASeTP	Natural	7.93 ± 0.14 (SeTPS-1); 5.59 ± 2.28 (SeTPS-2); 6.00 ± 2.93 (SeTPS-3)	Se-O-c; O-Se-O; Se-O	Enhancement of intestinal barrier function; modulation of gut microbiota composition	[119]
Ziyang tea	Se-ZYTP	Natural	2.14	/	Antitumor activity	[117]
Tea	Se-TPS	Natural	1.987 (Se-TPS); 0.97 (Se-TPS1); 0.44 (Se-TPS2); 0.34 (Se-TPS3)	/	Antioxidant activity	[114]
Tea	Se-TPS	Natural	2.76 ± 0.10	/	Antitumor activity; Immunomodulatory activity	[115]
Tea	SeTPS-1; SeTPS-2	Natural	23.50 (SeTPS-1); 13.47 (SeTPS-2)	O-Se-O; Se=O	Reduction of H_2_O_2_-induced DNA damage	[120]
*Rosa laevigata*	Se-RLFPs	Natural	16.49 (Se-RLFP-I); 21.61 (Se-RLFP-II)	Se-O-C; Se=O	Neuroprotective effects	[121]
pennycress (*Thlaspi arvense* L.)	Se-PPSs	Natural	13.56 ± 1.87 (Se-PPS1; 15.36 ± 2.30 (Se-PPS3)	Se=O; O-Se-O;	Antioxidant activity	[122]
Yam (*Dioscorea opposita* Thunb.)	SeYPS-1, SeYPS-2	Artificial se-enriched	715 (SeYPS-1); 1545 (SeYPS-2)	/	Immunomodulatory activity	[123]
*Rehmannia glutinosa*	/	Artificial se-enriched	2239	Se=O; Se-C	Immunomodulatory activity	[124]
*Ulmus pumila* L.	Se-PPU	Artificial se-enriched	/	/	Anti-inflammatory activity	[125]
*Castanea mollissima* Blume	sCPA	Artificial se-enriched	573.9	Se-O-C	Antitumor activity	[126]
*Glycyrrhiza uralensis*	SeGUP	Artificial se-enriched	1339	O-Se-O;	Antioxidant activity	[127]
*Artemisia sphaerocephala*	SeASP	Artificial se-enriched	22,400	Se=O; C-O-Se	Antitumor activity	[128]
Garlic	sGPSs	Artificial se-enriched	NA-SS: 29,400 (sGPS_8_); GA-SA: 26,300 (sGPS_9_); GA-SS: 10,500 (sGPS_2_); SOC: 9200 (sGPS_9_)	OSeC; SeO	Immunostimulatory activity	[129]
Garlic	sGPSs	Artificial se-enriched	10.53~38.27 (sGPS_1–9_)	Se = O; Se-O- c	Antioxidant activity; immunomodulatory activity	[130]
Sweet Potato	Se-SWP	Artificial se-enriched	12,740	C−O−Se; Se=O	Antioxidant activity; anticancer activity; antidiabetic activity	[131]
Alfalfa roots	Se-RAPS-2	Artificial se-enriched	320	C-O-Se; Se=O	Antioxidant activity; anticancer activity	[132]
Sweet corncob	Se-SCP	Artificial se-enriched	/	C-O-Se; Se=O	Antidiabetic activity	[133]

**Table 4:** Sources and biological activity of selenium-enriched polyphenols in plants.

Plant Source	Types of polyphenols	Se Source	Se Bond	Se Content (µg/L^−1^)	Biological Effects	Reference
Green tea	Flavonoids	Natural	Se=O; Se-O-C; An esterification reaction between the 3-–OH group and hydrogen selenite (HSeO_3_^−^).	15,690.4	Immunomodulatory activity; anti-inflammatory activity	[145]
*Ampelopsis grossedentata*	Dihydromyricetin	Artificial se-enriched	O-Se-O	/	Blood glucose-lowering effect; antitumor activity	[150]
/	Chrysin, Quercetin	Artificial se-enriched	Se=C	/	Anticancer activity	[149]
/	Quercetin	Artificial se-enriched	Se- o	/	/	[152]
/	Vitamin P	Artificial se-enriched	O-Se-O	/	Antitumor activity	[151]

## Data Availability

No new data were created or analyzed in this study.
